# Leptospirosis-Associated Acute Respiratory Distress Syndrome (ARDS) in Pregnancy: A Rare Presentation

**DOI:** 10.7759/cureus.61809

**Published:** 2024-06-06

**Authors:** Shivani Singh, Neema Acharya, Megha Karnik, Bingu Shiv kiran Reddy, Tejal Waghe, Aishwarya Beedkar

**Affiliations:** 1 Obstetrics and Gynecology, Jawaharlal Nehru Medical College, Datta Meghe Institute of Higher Education and Research, Wardha, IND; 2 Obstetrics and Gynaecology, Jawaharlal Nehru Medical College, Datta Meghe Institute of Higher Education and Research, Wardha, IND; 3 Pulmonary and Critical Care Medicine, Jawaharlal Nehru Medical College, Datta Meghe Institute of Higher Education and Research, Wardha, IND

**Keywords:** multidisciplinary management, neonatal complications, emergency cesarean section, acute respiratory distress syndrome (ards), pregnancy, leptospirosis

## Abstract

Leptospirosis, a zoonotic disease caused by spirochetes of the genus Leptospira, poses unique challenges in pregnancy due to its varied clinical presentation and potential adverse outcomes for both mother and fetus. We present a case of a 24-year-old primigravida at 35 weeks of gestation who presented with fever, dyspnea, and abdominal pain, and was ultimately diagnosed with leptospirosis complicated by acute respiratory distress syndrome (ARDS). Prompt initiation of antibiotic therapy, supportive care, and timely delivery via emergency cesarean section led to favorable maternal and neonatal outcomes. This case report underscores the importance of considering leptospirosis in pregnant patients presenting with similar symptoms, particularly in endemic regions, and highlights the critical role of multidisciplinary management in optimizing outcomes.

## Introduction

Leptospirosis, caused by pathogenic spirochetes of the genus Leptospira, is a zoonotic disease with a global distribution prevalent in urban and rural settings. The disease is transmitted to humans through direct or indirect contact with urine from infected animals, particularly rodents, which serve as reservoir hosts for the bacteria [1]. Leptospirosis has a broad spectrum of clinical manifestations, ranging from mild febrile illness to severe pulmonary hemorrhage, renal failure, and multiorgan dysfunction syndrome [2]. Pregnancy, a state characterized by altered immune function and physiological changes, poses unique challenges in the diagnosis and management of infectious diseases, including leptospirosis. While relatively rare, leptospirosis in pregnancy can have severe implications for both the mother and the fetus [3]. Pregnant women may present with nonspecific symptoms such as fever, myalgia, and headache, which can mimic other common obstetric conditions [4]. Furthermore, leptospirosis during pregnancy has been associated with adverse fetal outcomes, including intrauterine growth restriction, prematurity, and fetal demise [5].

The diagnosis of leptospirosis in pregnancy can be challenging due to overlapping clinical features with other common obstetric conditions and limitations in diagnostic testing. Serological tests, such as enzyme-linked immunosorbent assay (ELISA) and microscopic agglutination test (MAT), are commonly used to diagnose leptospirosis. Still, their interpretation during pregnancy may be complicated by physiological changes in immune function and cross-reactivity with other pathogens [6]. Additionally, there is limited data on the safety and efficacy of antimicrobial therapy in pregnant women with leptospirosis, necessitating careful consideration of the risks and benefits of treatment options [7]. Given the potential for severe maternal and fetal complications, prompt recognition and management of leptospirosis in pregnancy are paramount. Multidisciplinary collaboration involving obstetricians, infectious disease specialists, and neonatologists is essential for optimizing maternal and neonatal outcomes in these patients [8].

## Case presentation

A 24-year-old woman, primigravida, currently at 35 weeks of gestation, presented to the emergency department of our tertiary care center with a constellation of symptoms. She complained of experiencing low-grade fever and chills over the past three days. Additionally, she reported progressive dyspnea upon exertion. The patient described colicky abdominal pain radiating to her thigh and back. Physical examination revealed tachypnea, dyspnea, and grade 2 pitting pedal edema. Auscultation of the lungs detected bilateral crepts in the lower lobes.

Upon initial assessment, her vital signs were notable for a pulse rate of 114 beats per minute, a body temperature of 100.4 °F, and a blood pressure of 140/90 mmHg in the left lateral supine position. Oxygen saturation was measured at 92%. There were no remarkable findings on cardiovascular examination, although the patient appeared pale without evidence of icterus. Generalized myalgia was also noted. Abdominal examination revealed a uterus consistent with 34 weeks gestation, with normal and rhythmic fetal heart sounds above 144 beats per minute. The cervix was closed and uneffaced. Minor hematuria was observed.

**Figure 1:**
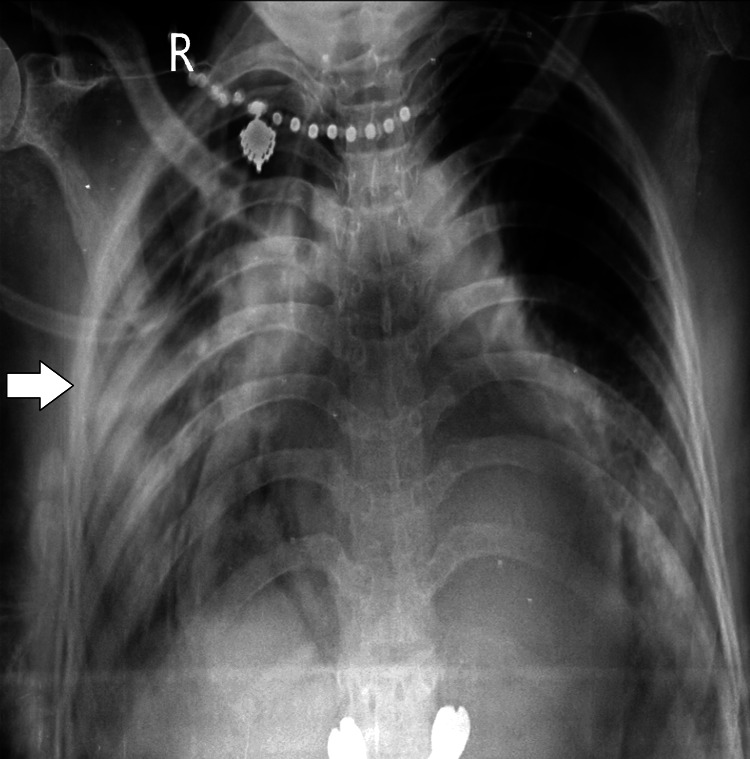
Chest X-ray posteroanterior (PA) view The image reveals consolidation in the right middle and lower lobes, as well as consolidation in the left lower lobe of the lung

Following the initial assessment, routine investigations and specific tests were ordered to ascertain the etiology of the patient's symptoms. Laboratory findings revealed leukocytosis (18,000/L), thrombocytopenia (platelets: 1.5 L), and decreased hematocrit (27%). Elevated inflammatory markers were evident, with C-reactive protein levels measuring 12 mg/dL. Renal function tests showed a BUN of 18 mg/dL and a creatinine level of 0.8 mg/dL. Liver function tests indicated mild hepatocellular injury, with elevated transaminases and alkaline phosphatase within ranges suggestive of hepatocellular involvement. A total bilirubin of 15 mg/dL, with a direct component of 5 mg/dL, was also observed. Arterial blood gas analysis revealed a pH of 7.38, HCO_3_ of 24 mEq/L, PaCO_2_ of 38 mmHg, and PaO_2_ of 80 mm Hg. Chest X-ray showed consolidation in the right middle and lower lobe and consolidation in the left lower lobe of the lung (Figure 1).

Given the patient's clinical presentation and laboratory findings, concerns for leptospirosis were raised. Further testing confirmed a positive leptospira IgM test. Additionally, urinalysis demonstrated proteinuria with a +1 dipstick result. Management was initiated promptly with intravenous antibiotics, including ceftriaxone, and supportive measures, such as mechanical ventilation to maintain adequate oxygenation and hydration, were taken.

Ultrasonography revealed a single intrauterine live fetus corresponding to an average gestational age of 37 weeks, with normal Doppler flow and spectral waveform. With antibiotic coverage for seven days, the patient was deemed stable for emergency cesarean section. A male neonate weighing 2.5 kg was delivered via vertex presentation. The neonate was admitted to the neonatal intensive care unit (NICU) due to respiratory distress but tested negative for leptospirosis. Maternal breastfeeding was deferred until the patient tested negative for leptospira IgM. Both mother and neonate were discharged in good condition on days 10 and 5 post-cesarean section, respectively.

## Discussion

Leptospirosis, a zoonotic disease caused by spirochetes of the genus Leptospira, is characterized by a broad spectrum of clinical manifestations, ranging from mild flu-like symptoms to severe pulmonary hemorrhage and multiorgan dysfunction syndrome [9]. While leptospirosis is endemic in tropical and subtropical regions, cases have been reported worldwide, with seasonal variations influenced by climate and environmental factors [10]. Pregnancy presents unique challenges in the diagnosis and management of leptospirosis due to physiological changes and potential adverse effects on both maternal and fetal health. Our case illustrates the importance of considering leptospirosis as a differential diagnosis in pregnant patients presenting with fever, dyspnea, and abdominal pain, particularly in endemic areas [11]. Early recognition of the disease is crucial for the timely initiation of antibiotics and supportive care to prevent complications such as acute respiratory distress syndrome (ARDS) and adverse pregnancy outcomes [12].

ARDS is a rare but severe complication of leptospirosis, characterized by diffuse alveolar damage leading to hypoxemia and respiratory failure [13]. Prompt institutions of mechanical ventilation and supportive measures, as demonstrated in our case, are essential for improving maternal outcomes and reducing the risk of maternal mortality [14]. Close monitoring of maternal vital signs, fluid balance, and oxygenation status is necessary to guide appropriate management strategies and optimize outcomes [15]. Antibiotic therapy remains the cornerstone of treatment for leptospirosis, with penicillins and third-generation cephalosporins being the preferred agents [16]. In our case, intravenous ceftriaxone was initiated based on antimicrobial susceptibility patterns and current guidelines for managing severe leptospirosis in pregnancy [17]. Although antibiotic therapy effectively clears leptospiral infection and prevents complications, the optimal duration and choice of antibiotics remain areas of debate and require further research [18].

The management of leptospirosis in pregnancy should also take into account fetal well-being and neonatal outcomes. Maternal-fetal transmission of leptospires is rare, but adverse outcomes such as fetal demise, preterm birth, and neonatal leptospirosis have been reported [19]. In our case, an emergency cesarean section was performed after seven days of antibiotic coverage to minimize the risk of vertical transmission and ensure optimal maternal and neonatal care [20]. The neonate, despite experiencing respiratory distress requiring NICU admission, tested negative for leptospirosis and responded well to supportive measures, highlighting the importance of early detection and management in preventing vertical transmission and neonatal complications [21].

## Conclusions

Our case highlights the importance of considering leptospirosis as a potential cause of ARDS in pregnant women, especially in regions where the disease is endemic. Early recognition, prompt initiation of appropriate antibiotic therapy, and supportive management are critical for improving maternal and neonatal outcomes. Collaboration among obstetricians, infectious disease specialists, and neonatologists is essential for comprehensive care in such complex cases. Further research is needed to elucidate optimal management strategies and long-term outcomes in pregnant women with leptospirosis. Heightened awareness, timely intervention, and multidisciplinary care are paramount in ensuring favorable outcomes for the mother and her newborn.
